# Tissue-specific biochemical differences between chronic wasting disease prions isolated from free-ranging white-tailed deer (*Odocoileus virginianus*)

**DOI:** 10.1016/j.jbc.2022.101834

**Published:** 2022-03-16

**Authors:** Kaitlyn Wagner, Robyn Pierce, Elizabeth Gordon, Arielle Hay, Avery Lessard, Glenn C. Telling, Jennifer R. Ballard, Julie A. Moreno, Mark D. Zabel

**Affiliations:** 1 Prion Research Center, College of Veterinary Medicine and Biomedical Sciences, Colorado State University, Fort Collins, Colorado, USA; 2 Department of Microbiology, Immunology and Pathology, College of Veterinary Medicine and Biomedical Sciences, Colorado State University, Fort Collins, Colorado, USA; 3 Research Division, Arkansas Game and Fish Commission, Little Rock, Arkansas, USA; 4 Department of Environmental and Radiological Health Sciences, College of Veterinary Medicine and Biomedical Sciences, Colorado State University, Fort Collins, Colorado, USA

**Keywords:** prions, chronic wasting disease, strains, protein structure, protein conformation, brain, lymph node, CNS, central nervous system, CWD, chronic wasting disease, LN, lymph node, PK, proteinase K, PMCA, protein misfolding cyclic amplification, rpu, relative PMCA units

## Abstract

Chronic wasting disease (CWD) is an invariably fatal prion disease affecting cervid species worldwide. Prions can manifest as distinct strains that can influence disease pathology and transmission. CWD is profoundly lymphotropic, and most infected cervids likely shed peripheral prions replicated in lymphoid organs. However, CWD is a neurodegenerative disease, and most research on prion strains has focused on neurogenic prions. Thus, a knowledge gap exists comparing neurogenic prions to lymphogenic prions. In this study, we compared prions from the obex and lymph nodes of naturally exposed white-tailed deer to identify potential biochemical strain differences. Here, we report biochemical evidence of strain differences between the brain and lymph node from these animals. Conformational stability assays, glycoform ratio analyses, and immunoreactivity scanning across the structured domain of the prion protein that refolds into the amyloid aggregate of the infectious prion reveal significantly more structural and glycoform variation in lymphogenic prions than neurogenic prions. Surprisingly, we observed greater biochemical differences among neurogenic prions than lymphogenic prions across individuals. We propose that the lymphoreticular system propagates a diverse array of prions from which the brain selects a more restricted pool of prions that may be quite different than those from another individual of the same species. Future work should examine the biological and zoonotic impact of these biochemical differences and examine more cervids from multiple locations to determine if these differences are conserved across species and locations.

Chronic wasting disease (CWD) is unique among prion disease as the only prion disease known to infect and be naturally transmitted between both captive and free-ranging populations. Mathematical models indicate that direct and indirect horizontal transmission of CWD is the most prevalent form of transmission (1, 2, 3, 4, 5), but vertical transmission also contributes to CWD transmission (6, 7, 8). Infectious prions have been detected in excreted bodily fluids including saliva, urine, and feces, as well as antler velvet, blood, and reproductive tissues (6, 9, 10, 11, 12). While CWD causes a neurological disease, CWD prions are profoundly lymphotropic, and these peripheral prions are the most likely to shed into the environment and contribute to horizontal and vertical disease transmission (13, 14, 15, 16, 17). Thus, it is critical to determine if any unique characteristics of extraneural prions exist that affect CWD pathogenesis and transmission.

While all prion diseases result from a misfolding of the normal host protein, PrP^C^, to a misfolded form, PrP^Sc^, different biochemical characteristics and disease phenotypes suggest a phenomenon of prion strains transmitting distinct disease characteristics epigenetically enciphered within unique prion structures (18, 19, 20). Thus, different prion strains sometimes have significant strain differences. There are multiple CWD strains that have been identified from North American isolates, including CWD-1, CWD-2, H95+, and Wisc-1 (21, 22). Importantly, the H95+ strain has been shown to emerge from white-tailed deer (*Odocoileus virginianus*) that have the more resistant genotype, 96SS, and this strain has been demonstrated to have an expanded host range, highlighting the importance of continued strain characterization (22). CWD-resistant *PRNP* polymorphisms have emerged in wild elk populations, like Rocky Mountain Elk (*Cervus canadensis nelsoni*), but it is unclear how these polymorphisms may affect novel and/or atypical prion strain emergence in these population (23); as happened with the emergence of the Nor98 scrapie strain in sheep expressing classical scrapie-resistant genotypes (24). Selection of CWD-resistant genotypes in white-tailed deer may also be occurring (25). These data highlight the necessity of continued strain identification and characterization from multiple sources. Understanding strain differences and potentially different transmission dynamics is of critical importance to understand CWD and control its spread.

Of particular concern, extraneural prions have been shown to have increased zoonotic potential (26). This has important implications for cross species transmission and risk for humans potentially contracting CWD from eating infected skeletal muscle or while cleaning a deer in the field (27). While no evidence currently supports natural xenotransmission of CWD prions from cervids to other animal species, CWD prions are infectious to cattle (28), sheep (29), swine (30), and cats (31) when experimentally inoculated intracerebrally. Cattle and cats were resistant to CWD infection after oral exposure, but pigs were susceptible at low levels (30). These data suggest that there is a risk of transmission to additional species and populations, warranting continued monitoring and surveillance of CWD prion strains.

Most of the CWD and prion research completed to date focused on brain-derived prions, likely because prion diseases are neurodegenerative, brain samples are easy to work with and contain the highest titers of prions in infected animals. However, prions shed into the environment likely are extraneural prions, such as those replicated within lymph nodes (LNs). Far less is known about the transmissibility of these peripheral prions, but research suggests LN-derived prions have similar titers to brain-derived prion titers on transgenic mouse bioassay (32). Furthermore, numerous immune receptors and different proteins involved in the complement cascade have been shown to influence prion strain selection, implicating the immune system as an important player in prion strain selection (33, 34, 35, 36, 37, 38, 39). This research suggests that lymphogenic prions likely exhibit more strain diversity than neurogenic prions (40, 41). Tissue-specific differences in strain heterogeneity, as reflected in the prion cloud hypothesis, predict different prion strains with different biochemical and structural characteristics in LNs than in the brain (40). Therefore, any differences between the brain-derived and LN-derived prions must be investigated to aid our understanding of intrahost and interspecies prion dynamics.

Based on current knowledge of CWD transmission, prion strain selection, and differential interspecies transmission, we hypothesize that LNs replicate more diverse CWD prion strains than the brain within and among individuals. While extensive research has focused on brain samples from cervid and transgenic mouse brains, less research has been dedicated to studying and characterizing peripheral prions, leaving a critical knowledge gap that this work addresses. Furthermore, very little work has characterized structural differences between brain- and LN-derived prions from a natural host prior to passage to transgenic mice or other model organisms. While bioassay is a central pillar to prion biology and strain characterization, there are other host factors and transgene expression level differences that influence strain emergence, emphasizing the importance of assessing strain characteristics of prions isolated from the natural host (42, 43).

For this study, we assessed biochemical strain differences between paired obex and LN samples from naturally exposed white-tailed deer from Arkansas, USA. These analyses reveal significant differences between brain-derived and LN-derived prion isolates in some of our biochemical assays. While we observed no conformational stability differences between brain- and the LN-derived prions, we observed electrophoretic differences and statistically significant differences in the glycoform ratio of PrP^Sc^ from brain compared to LN samples. Lymphogenic prions exhibited greater overall variance in mean glycoform ratios and conformational stability than neurogenic prions. Surprisingly, we observed greater biochemical differences among brain-derived prions than LN-derived prions across individuals. These data lead us to propose a mechanism whereby the lymphoreticular system propagates a diverse array of prions from which the brain selects a more restricted pool of prions that may be quite different than those from another individual of the same species. Assessing differences in biochemical signatures between prions from brain and LNs among individuals will inform future studies poised to assess biological differences, including zoonotic potential, between neurogenic and lymphogenic prions using bioassay and other traditional prion assays.

## Results

### Sample origin, preparation, and result overview

Samples used in this study were all collected from naturally exposed white-tailed deer in the state of Arkansas and shared with us from our collaborators from the Arkansas Fish and Game Commission. *PRNP* gene sequencing revealed that all deer shared the same amino acid sequences at known CWD susceptibility loci (95QQ, 96GG, 116AA, 132MM, 225SS, 226QQ). All deer also harbor similar prion titers as determined by two distinct *in vitro* assays [(44, 45); cervid prion cell assay and protein misfolding cyclic amplification, Table 1].

**Table 1:** Animals used in this study

*Sample*	Age (y)	Sex	Obex				Lymph node			
			*CSA*	*Glycoform ratio*	*Prion titer*		*CSA*	*Glycoform ratio*	*Prion titer*	
					*CPCA (mean ± 95% CI log*_*10*_*LD*_*50*_*/g)*	*PMCA (mean ± 95% CI rpu)*			*CPCA (mean ± 95 CI log*_*10*_*LD*_*50*_*/g)*	*PMCA (mean ± 95 CI rpu)*
10023	2.5	F	Yes	Yes	6.6 ± 0.5	100 ± 2	Yes	Yes	3.0 ± 0.2	33 ± 11
10074	5.5+	F	Yes	Yes	6.3 ± 0.6	89 ± 13	Yes	Yes	3.3 ± 0.3	47 ± 17
10083	1.5	M	Yes	Yes	6.0 ± 0.3	89 ± 13	Yes	Yes	3.1 ± 0.3	41 ± 15
07399	5.5+	F	Yes	Yes	6.1± 0.2	100 ± 2	Yes	Yes	3.2 ± 0.1	44 ± 13
07416	3.5	M	No	No	5.8 ± 0.8	78 ± 12	Yes	Yes	3.0 ± 0.2	33 ± 11
10030	3.5	M	No	No	5.9 ± 0.7	78 ± 12	Yes	Yes	3.3 ± 0.4	57 ± 9
14707	4.5	M	Yes	Yes	6.3 ± 0.2	100 ± 2	No^a^	No^a^	1.8 ± 0.5^b^	11 ± 7^b^
10080	2.5	M	No	No	5.0 ± 0.3	63 ± 18	No	No	2.4 ± 0.3	22 ± 17
07415	2.5	M	No	No	4.7 ± 0.4	67 ± 2	No	No	1.2 ± 0.7^b^	11 ± 7^b^

Abbreviations: CSA, conformational stability assay; CPCA, cervid prion cell assay; PMCA, protein misfolding cyclic amplification; rpu, relative PMCA units.

a Excluded from analyses because only one of five western blots of these samples gave interpretable data, resulting in too few replicates for conformational stability analysis or glycoform ratio to be determined. We performed pairwise analyses of only those samples that yielded reproducibly interpretable results.

b *p* < 0.05, compared to all other LN titers except 10080 and each other.

We optimized our assays to obtain the clearest, most reproducible data possible. If we employed the same proteinase K (PK) digestion and Western Blot methods for brain/obex samples and LN samples, brain-derived PrP^Sc^ signals were indistinguishable from PrP^C^ signals. We therefore optimized digestion conditions for each tissue type. Brain samples required PK digestion in the presence of 1% Triton-X 100, with a higher concentration of PK (100 μg/ml), and less starting total protein (5% w/v homogenate before PK digestion) electrophoresed through the gel (data not shown). LN samples ran well when more protein was loaded onto the gel (10% w/v starting homogenate) and digested with less PK (50 μg/ml) than obex samples. Of the nine animals that had paired obex and LN samples, only four of the animals gave us interpretable data from both the tissues that enabled us to compare intrahost variation (Table 1).

### Conformational differences between obex and LN samples at ≥ 2.5 M GdnHCl

**Figure 1:**
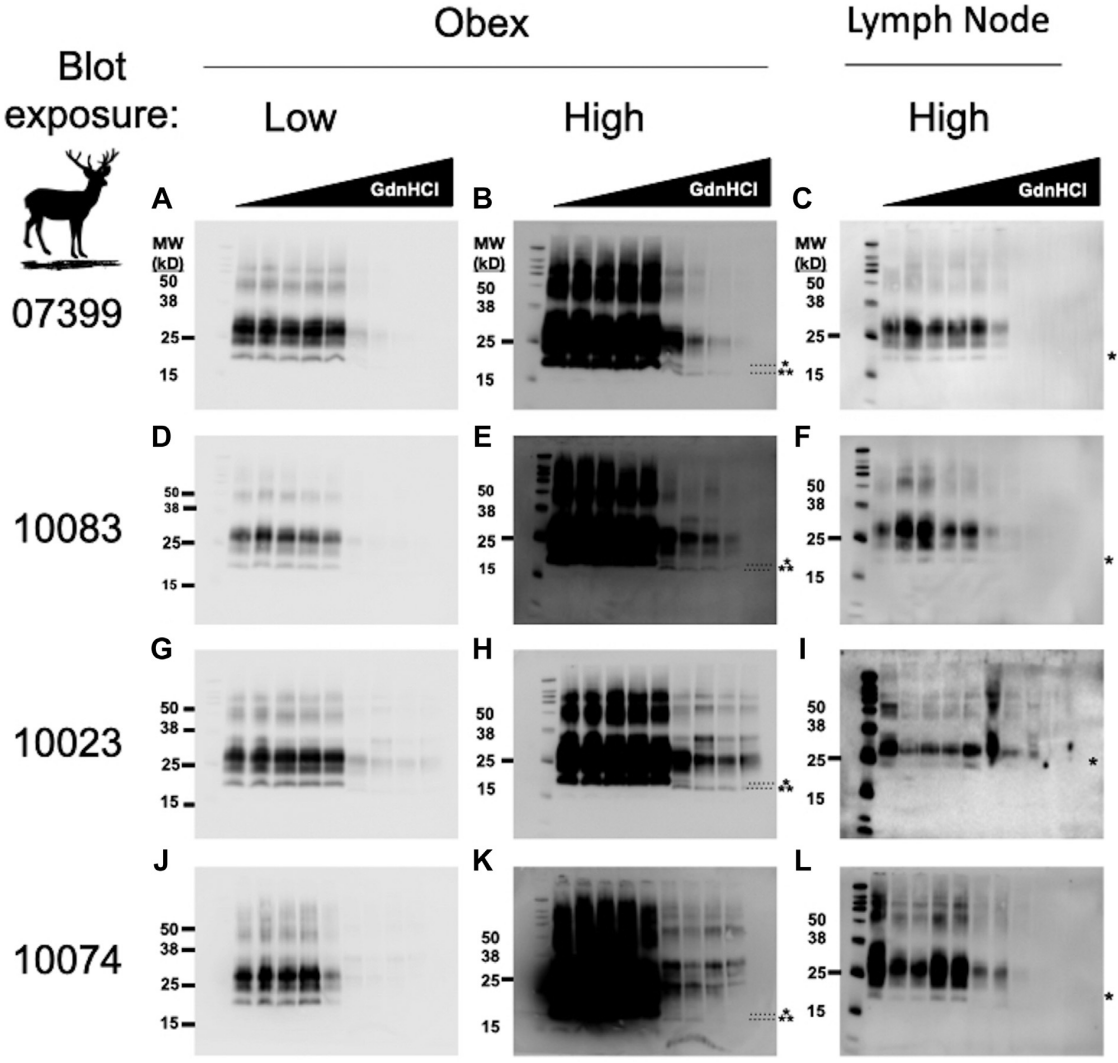
**Obex prions adopt an alternative conformation in the presence of ≥ 2.5 M GdnHCl compared to lymph node prions from the same animal.** Western blots to the right of the animal identification number are all from the same individual. Sample obex blots imaged at a typical exposure (panels *A*, *D*,*G*,*J*) and overexposed (*B*,*E*,*H*,*K*) reveal a unique, faster-migrating PrP^Sc^ electrophoretic signature in obex samples (note the unglycosylated 19kD band∗ at < 2.5 M GdnHCl compared to a 17 kD band∗∗ at ≥ 2.5 M GdnHCl) absent in PrP^Sc^ from lymph node samples (*C*,*F*,*I*,*L*). Markers to the *right of blots* indicate the molecular weight (MW) in kilodaltons (kD). Other bands from the protein MW ladder, from top to bottom, indicate the 260, 125, 90, 70, 50, 38, 25, 15, and 8 kD (visible only in lymph node blots in panels *C*,*F*,*I*,*L*).

Samples were prepared for analysis by conformational stability and glycoform ratio as described in the methods section. Samples were then run on a Western blot to collect densitometric and electrophoretic mobility data. Differences in electrophoretic mobility of a prion sample reveal structural differences that dictate PK accessibility, resulting in different PK-resistant core fragments of PrP^Sc^. These heritable structural differences are reliable biochemical indicators of different prion strains (46, 47, 48, 49, 50). Obex samples that were incubated in 2.5 M GdnHCl and greater migrated faster than samples exposed to lower concentrations of GdnHCl (Fig. 1). One LN sample may have exhibited this 17 kD band (Fig. 1*L*), but this was not consistently observable, and the lower migrating band may be due simply to a slight tilt in the WB image. This 17 kD band was only observed consistently in obex samples, and these data were consistent among all four individuals.

### Greater variability in conformational stability of LN prions compared to brain prions

**Figure 2:**
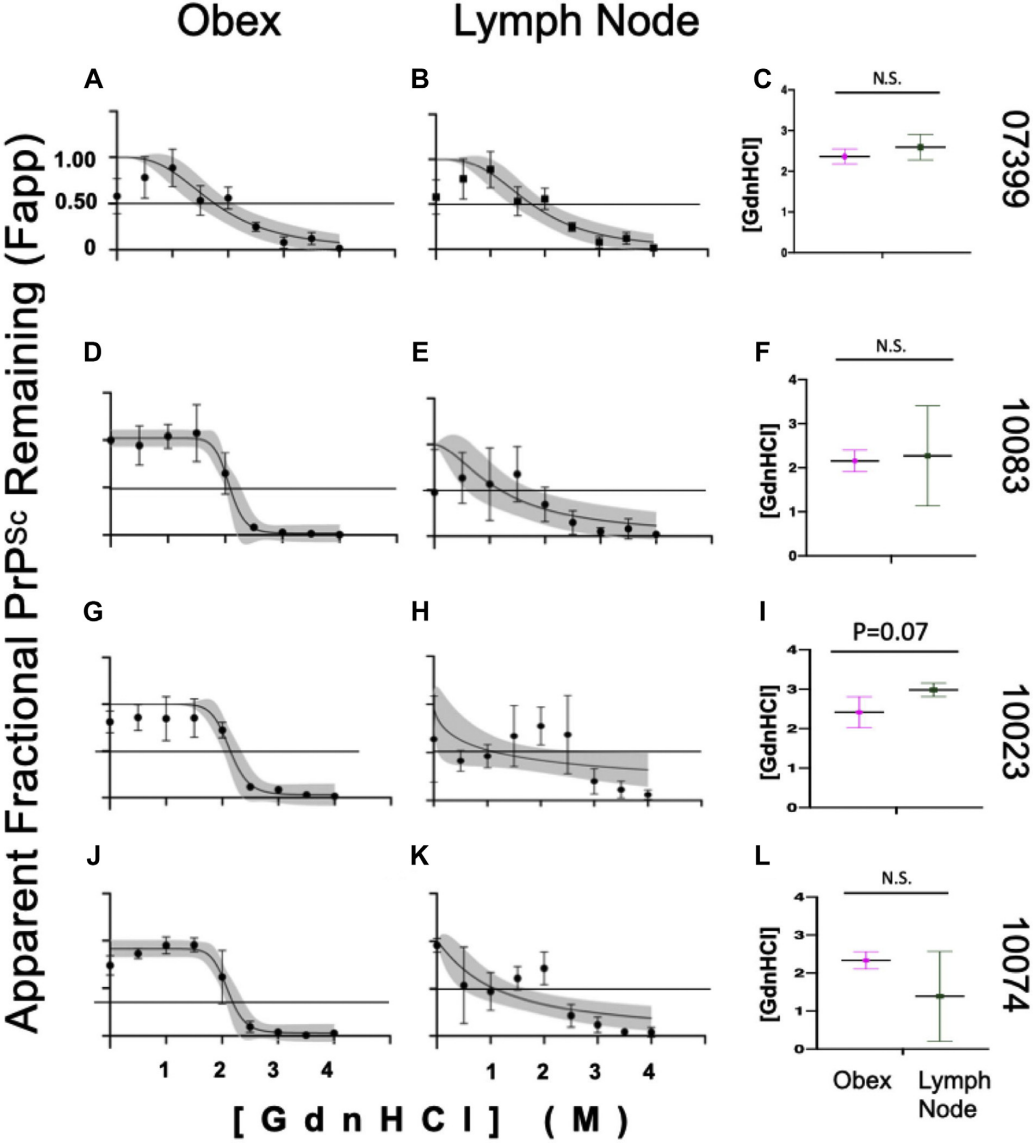
**No differences in conformational stability between obex- and lymph node-derived prions in paired samples from the same deer.** No differences were observed between obex and lymph node samples from deer 07399 (*A–C*), 10083 (*D–F*), 10023 (*G–I*), or 10074 (*J–L*). Samples were treated with GdnHCl as described in the methods section. Four-parameter sigmoidal curves are shown for both obex (*A*, *D*, *G*, *J*) and lymph node (*B*, *E*, *H*, *K*) samples and depict the best-fit curve and 95% confidence interval (CI) bands (*gray shading*) from at least three experiments. Plots show the mean and 95% CI of the samples at each concentration of GdnHCl. The horizontal line in each graph depicts 50% of the signal remaining compared to untreated samples, set at 100%. Panels *C*, *F*, *I*, and *L* depict the mean and 95% CI of the GdnHCl_1/2_ values from the individual replicates for both the obex and the lymph node of the same animal. While no statistical differences were found between sample means, the denaturation curves for brain samples were different, and better fit the data than the lymph node denaturation curves. The difference between obex and lymph node of sample 10023 is trending toward significance (*p* = 0.07). Unpaired *t* test, *p* < 0.05. N.S., not significant.

Distinct prion strains can have different conformational stability in the presence of chaotropic denaturing agents like GdnHCl. We compared conformational stability of prions isolated from LN to prions isolated from brain samples to determine if this strain characteristic would reveal potentially different strains from different tissues within the same animal. We treated samples with increasing concentrations of GdnHCl and determined their [GdnHCl]_1/2_ values, which is the [GdnHCl] that eliminates half of the PrP^Sc^ compared to the untreated sample. While one animal trended toward statistical significance, we detected no statistical differences in conformational stability measured between paired obex-derived and LN–derived prions in any of the four individuals examined (unpaired *t* test, *p* < 0.05, Fig. 2). However, we did observe a statistical difference in the variance of mean [GdnHCl]_1/2_ values between the obex and LN-derived prion samples from animal 10083 (F-test, *p* < 0.05). Also, brain denaturation curves fit the data better (R^2^ range from 0.8272–0.9242) than the denaturation curves for LN samples (0.1991–0.6113). Differences in mean [GdnHCl]_1/2_ variances for animal 10023 trended toward significance (F-test, *p* = 0.07). The other two samples did not exhibit significant difference in conformational stability variance (F-test, *p* > 0.05).

**Figure 3:**
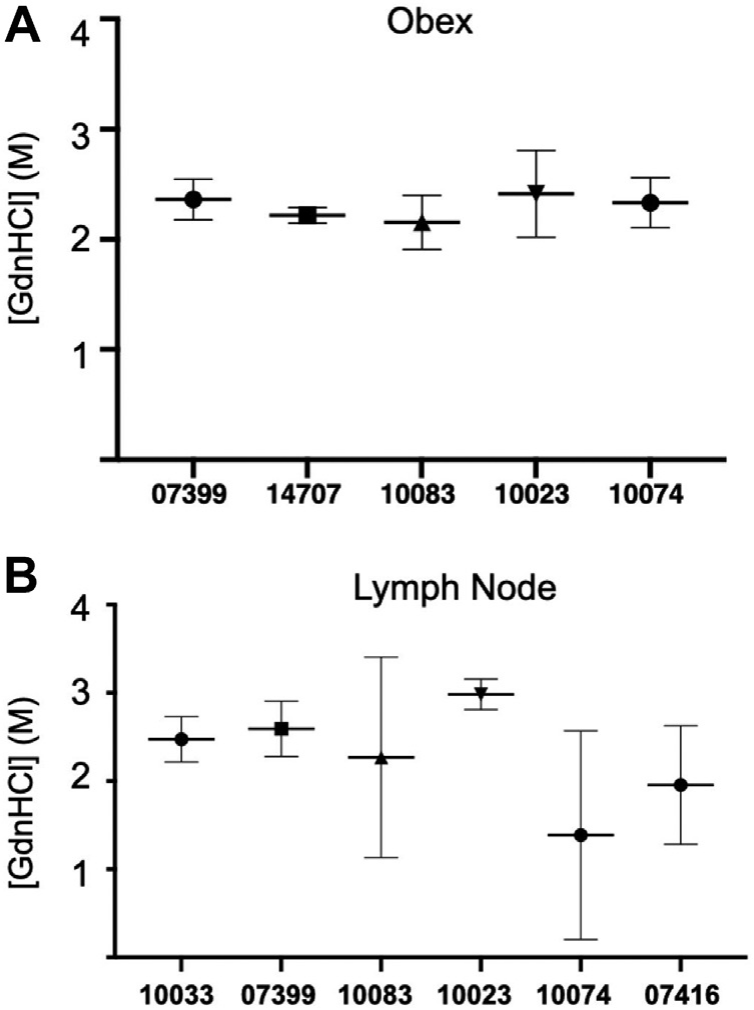
**No conformational stability differences among individuals in either obex or lymph node derived prions.** We observed no differences in mean [GdnHCl]_1/2_ values in prions isolated from (*A*) obex samples or (*B*) lymph nodes among any individuals. One-way ANOVA with Tukey adjustment (*p* > 0.05).

**Figure 4:**
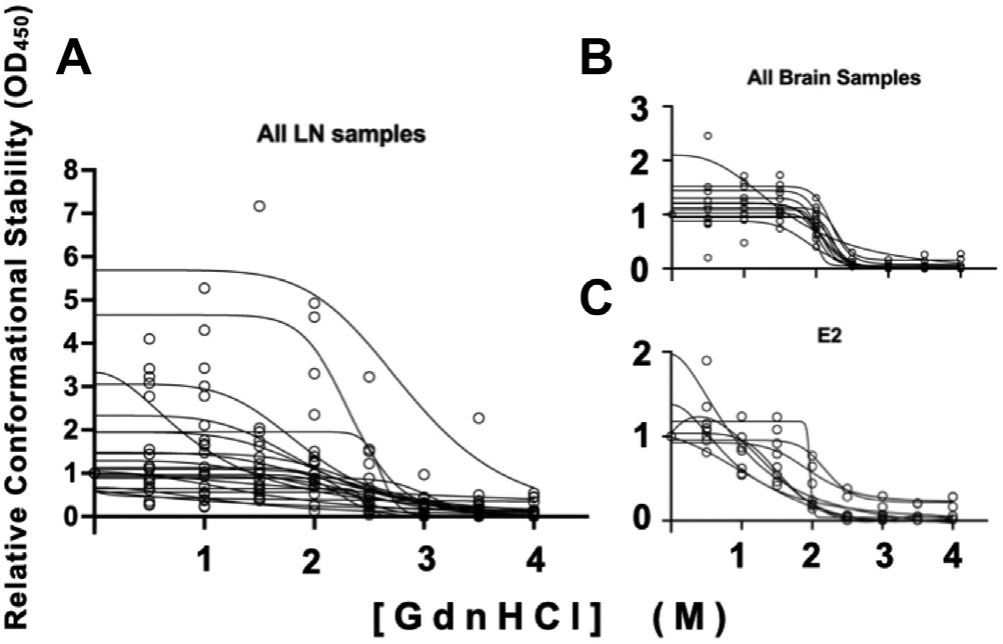
**Significant difference in variance of [GdnHCl]**_**1/2**_**values between LN and brain-derived prion isolates across individuals.** While we observed no statistical differences in mean [GdnHCl]_1/2_ values between LN and brain derived prion isolates (paired *t* test, *p* > 0.05), we found significant differences in the variance of these means (F test, *p* < 0.05). Conformational stability curves for each isolate is shown in triplicate. *A*, prions isolated from LNs exhibited increased variance compared to (*B*) prions isolated from brains and (*C*) an Elk brain used as a laboratory control (E2). LN, lymph node.

To assess potential differences in conformational stability among individuals in either the brain or the LN, we compared prions isolated from the same tissue across all individuals that yielded interpretable data, not just the four samples with paired obex and LN data that allowed for within animal comparison (Table 1). We observed no statistical differences in conformational stability (mean [GdnHCl]_1/2_) in prions derived from either brain or LN when compared among individuals (Fig. 3). To determine if significant biochemical differences exist between brain and LN prions generally across individuals, we analyzed mean [GdnHCl]_1/2_ values calculated from individual values aggregated for each tissue from all individuals. While we observed no significant differences in mean [GdnHCl]_1/2_ values from brain (1.9 M, 95% CI: 1.8–2M) and LN (2.2 M, 95% CI: 1.7–2.7 M; paired *t* test, *p* < 0.05), LN prion samples exhibited statistically more variance in mean [GdnHCl]_1/2_ than obex samples (Fig. 4, F-test, *p* < 0.05).

### PrP^Sc^ glycoform ratio differences between prions from paired brain and LNs in the same animal

**Figure 5:**
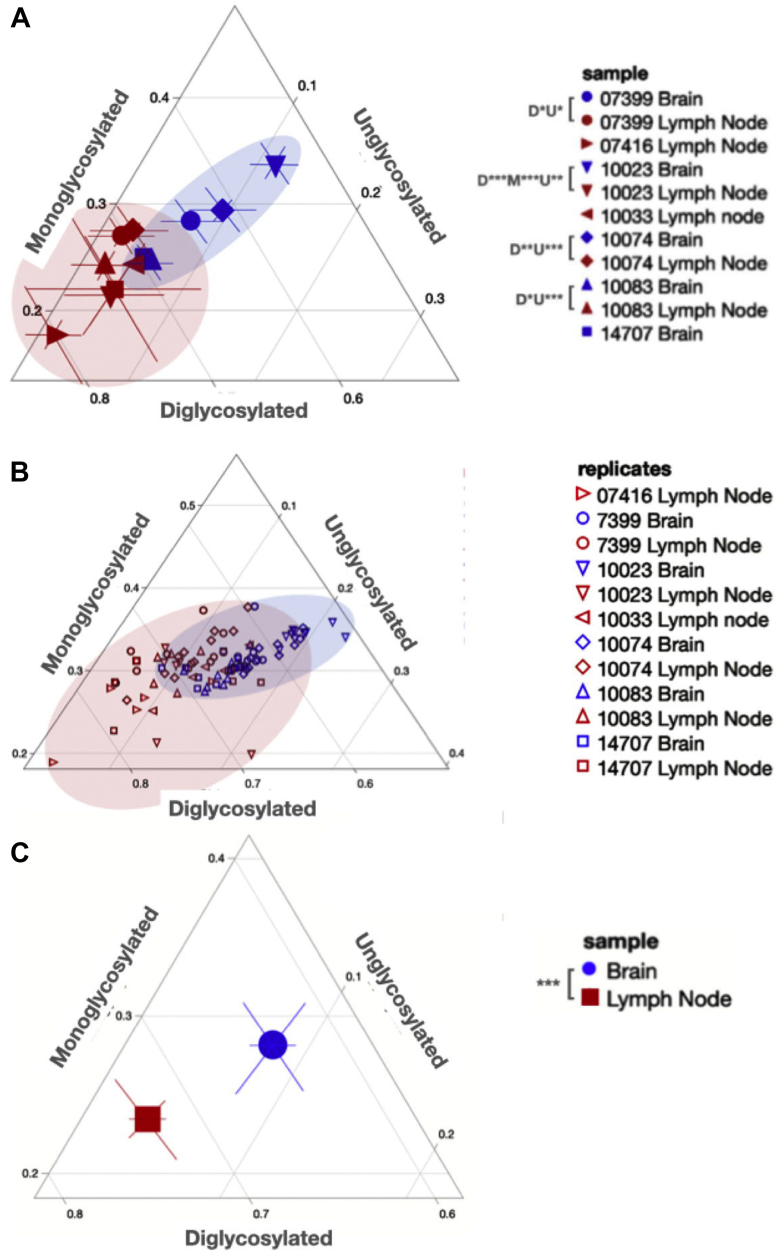
**PrP**^**Sc**^**glycoform ratio differences within and among deer tissue samples.** Ternary plots facilitate glycoform ratio comparisons. *A*, mean glycoform ratios with 95% CI shown for paired [07399 (*circles*), 10023 (*down triangles*), 10074 (*diamonds*), and 10083 (*up triangles*)] and unpaired samples from obex (Brain, *blue*) and lymph node (*red*). *p*-values are shown in the legend for diglycosylated (D), monoglycosylated (M), and unglycosylated (U) proportions. *p*-values for differences between individual deer are summarized in Tables 2 and 3. *B*, raw glycoform ratio data shown for all replicates from all tissues analyzed. Shaded areas depict the range of PrP^Sc^ glycoform ratios from both tissues. *C*, overall mean glycoform ratios with 95% CI for all brain (*blue circle*) and lymph node (*red square*) samples aggregated from individual glycoform ratios from all replicates from all animals shown in (*B*). See Figure 1 for representative Western blots from which we calculated these glycoforms ratios. ∗*p* < 0.05, ∗∗*p*< 0.01, ∗∗∗*p* < 0.001. One-Way ANOVA with Tukey adjustment.

Glycoform ratios are another heritable biochemical trait of prion strains and have been used in the characterization of the prion strains causing bovine spongiform encephalopathy, Creutzfeldt-Jakob Disease, and CWD (48, 49, 50, 51, 52). Just as we assessed conformational stability, we compared PrP^Sc^ glycoform ratios of prions in obex and LN tissue samples from the same deer to assess whether distinct prions may reside in distinct tissues within the same host. We found significant differences in proportions of at least two glycoforms between matched brain and LN samples for all four individuals examined (unpaired *t* test, *p* < 0.05, Fig. 5). When comparing glycoform ratios of prions in tissues across individuals, we observed few differences in glycoform ratio in LN prions (Fig. 5 and Table 2). However, we detected many glycoform ratio differences among obex prions across individuals. In fact, only two obex samples, when we compared their PrP^Sc^ glycoform ratios to each other, were not statistically different (ANOVA with Tukey adjustment; Fig. 5 and Table 3). Finally, when comparing mean glycoform ratios calculated from aggregated individual ratios for each tissue across all individual deer, we observed statistical differences in glycoform ratio across all three glycosylation states (paired *t* test, *p* < 0.001; Fig. 5*C*).

**Table 2:** PrP^Sc^ glycoform comparison in prions across all paired lymph node samples

Samples compared^a^	Diglycosylated		Monoglycosylated		Unglycosylated	
	*p* value	Significance	*p* value	Significance	*p* value	Significance
07399 *versus* 10083	0.9514	ns	0.9369	ns	0.9996	ns
07399 *versus* 10023	0.7472	ns	0.1899	ns	0.5652	ns
07399 *versus* 07416	0.0124	b	0.0107	b	0.9990	ns
07399 *versus* 10033	>0.9999	ns	0.9204	ns	0.2456	ns
07399 *versus* 10074	0.9979	ns	>0.9999	ns	0.8841	ns
10083 *versus* 10023	0.9991	ns	0.8572	ns	0.8754	ns
10083 *versus* 07416	0.2354	ns	0.2354	ns	0.9926	ns
10083 *versus* 10033	0.9900	ns	>0.9999	ns	0.5846	ns
10083 *versus* 10074	0.8418	ns	0.8949	Ns	0.9932	ns
10023 *versus* 07416	0.3617	ns	0.8279	ns	0.5411	ns
10023 *versus* 10033	0.9168	ns	0.8781	ns	0.9907	ns
10023 *versus* 10074	0.5528	ns	0.1598	ns	0.9798	ns
07416 *versus* 10033	0.0687	ns	0.2556	ns	0.2635	ns
07416 *versus* 10074	0.0065	b	0.0092	∗∗	0.8289	ns
10033 *versus* 10074	0.9964	ns	0.8735	ns	0.7688	ns

∗∗*p* < 0.01.

a One-way ANOVA with Tukey adjustment.

b significant, ns, not significant.

**Table 3:** PrP^Sc^ glycoform comparison in in prions across all paired obex samples

Sample comparison^a^	Diglycosylated		Monoglycosylated		Unglycosylated	
	*p* value	Significance	*p* value	Significance	*p* value	Significance
07399 *versus* 14707	0.0066	∗∗	0.0456	∗	0.0176	∗
07399 *versus* 10083	0.0173	∗	0.0251	∗	0.3450	Ns
07399 *versus* 10074	0.3187	ns	0.9244	Ns	0.0678	Ns
07399 *versus* 10023	<0.0001	∗∗∗∗	0.0011	∗∗∗	<0.0001	∗∗∗∗
14707 *versus* 10083	0.9968	ns	0.9993	Ns	0.6447	Ns
14707 *versus* 10074	<0.0001	∗∗∗∗	0.0051	∗∗	<0.0001	∗∗∗∗
14707 *versus* 10023	<0.0001	∗∗∗∗	<0.0001	∗∗∗∗	<0.0001	∗∗∗∗
10083 *versus* 10074	<0.0001	∗∗∗∗	0.0025	∗∗	0.0003	∗∗∗
10083 *versus* 10023	<0.0001	∗∗∗∗	<0.0001	∗∗∗∗	<0.0001	∗∗∗∗
10074 *versus* 10023	0.0022	∗∗	0.0119	∗	0.1001	Ns

∗*p* < 0.05, ∗∗*p* < 0.01, ∗∗∗*p* < 0.001, ∗∗∗∗*p* < 0.0001, ns, not significant.

a One-way ANOVA with Tukey adjustment.

### Conformation-dependent ELISA confirms conformational differences between lymphoid and obex prions

**Figure 6:**
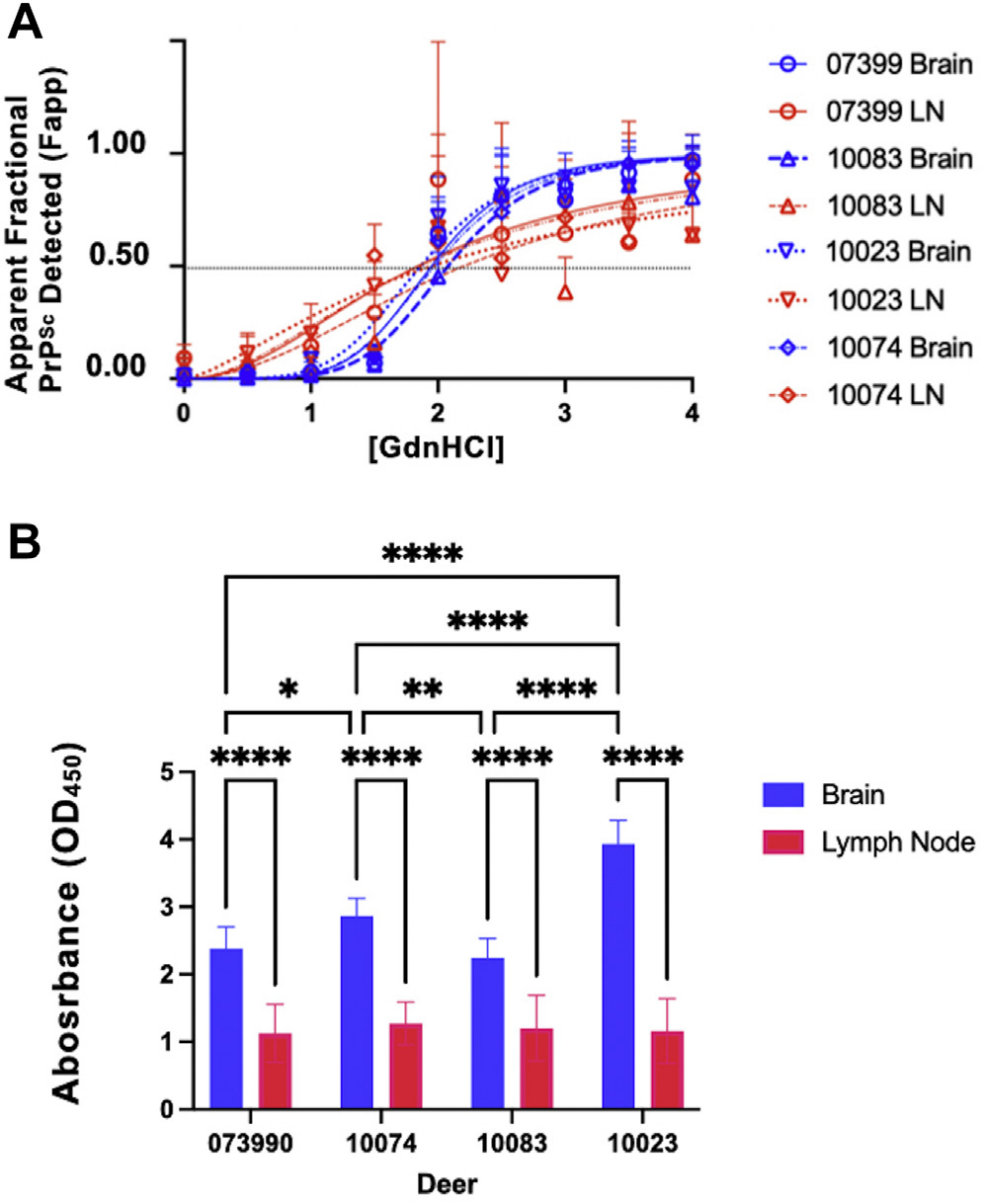
**7-5 ELISA reveals statistically different conformational stabilities in neurogenic prions from individual dear and greater variance in lymphogenic prion replicates.***A*, lymph node and obex samples were treated with increasing GdnHCl, then subjected to the 7-5 capture ELISA that preferentially identifies PrP^Sc^ conformations. We measured absorbance at 450 nm and fit denaturation curves. We observed statistically significant differences between LN and brain prion denaturation profiles and among the brain prion profiles (*p* < 0.05). *B*, 7-5 ELISA results for samples treated with 2M GdnHCl. We report means with 95% confidence intervals. We observed statistical differences in absorbance signals in brain prions across individual deer, but no statistically significant differences in lymph node prions from individual deer. We did observe greater variance in the mean absorbance of lymph node prions compared to brain prions.∗*p* < 0.05, ∗∗*p* < 0.01, ∗∗∗∗*p* < 0.0001. LN, lymph node.

We next address the possibility that greater background noise in our western blots combined with lower prion titers in lymphoid tissues accounted for the increased variance we observed in conformational stability and glycosylation between lymphogenic and neurogenic prions. The 7-5 ELISA is a conformation-based, capture ELISA that preferentially detects prion forms of PrP, while virtually eliminating the noise associated with PK digestion and Western blotting. Using this assay, we observed greater variance in our lymphoid samples and significant statistical differences among our obex samples from the four deer analyzed (Fig. 6). These data confirm our conformation and glycoform data demonstrating increased variance in lymphoid prions and emergence of unique brain-derived prions in individual deer.

### Immunoreactivity scanning reveals structural differences between neurogenic and lymphogenic prions

**Figure 7:**
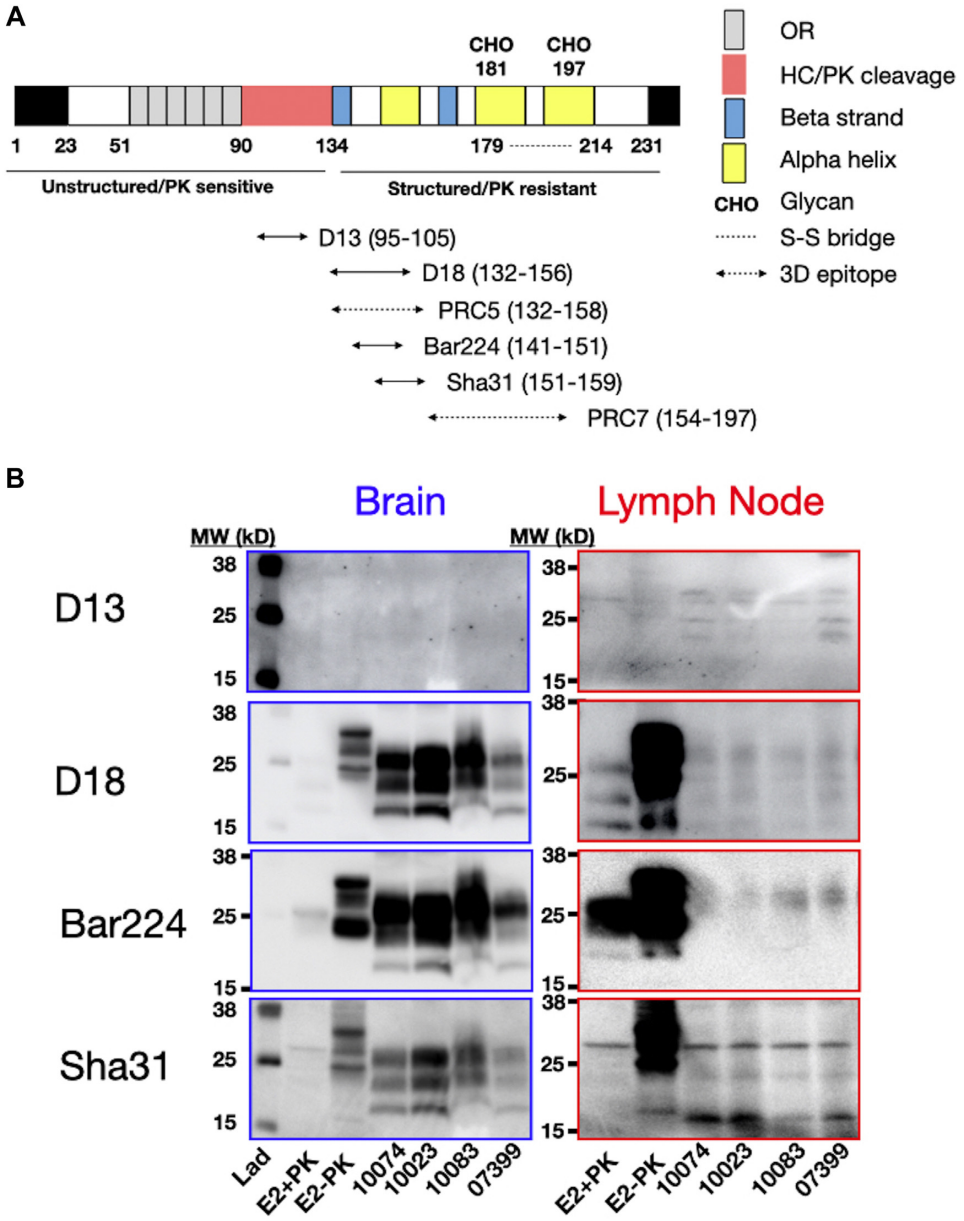
**Immunoreactivity scanning reveal structural differences between neurogenic and lymphogenic prions.***A*, schematic representation of the prion protein primary sequence with relevant structural features identified. *Double-ended arrows* below the schematic depict epitopes for the indicated antibodies used in (*B*) and in the 7-5 ELISA. *B*, brain and lymph node prion samples were PK digested (except E2-PK sample in lane three of brain blots and lane two of lymph node blots) and Western blotted using the indicated antibodies. Blots were probed, stripped, and re-probed with antibodies in order from top (D13) to bottom (Sha 31). Blots shown are representative of at least three experiments. Differential binding, electrophoretic mobility, and banding patterns reveal structural differences between lymphogenic and neurogenic prions and among neurogenic prions in individual cervid brains. Markers to the *right of blots* indicate the molecular weight (MW) in kilodaltons (kD). PK, proteinase K.

We also noted several differences in immunoreactivity when we probed prion samples with a panel of antibodies spanning residues 95 to 197 (Fig. 7). Specifically, antibody D13, whose epitope lies within the PK cleavage zone, recognized lymphogenic but not neurogenic prions, including our E2 control isolate. While lymphogenic prions exhibited nearly identical banding patterns across all antibodies used, neurogenic prions exhibited many differences in banding patterns and intensities across all antibodies.

## Discussion

CWD is an invariably fatal disease infecting cervids worldwide. This disease is devastating to the individual animals that become infected and has resulted in substantial population-level effects in free-ranging animals, including population decline and herd culling as a method of disease control (23, 53, 54, 55, 56, 57). The unique nature of prions and prion diseases, coupled with extremely facile animal-to-animal transmission, necessitates a thorough understanding of the pathogen causing disease.

Because CWD and other prion diseases are neurological, and the nervous system harbors the highest prion titers, most prion research has focused on brain-derived prions. While these pivotal studies are critical to our current understanding of prion disease, the profound lymphotropism and presence of infectious prions in extraneural sites should be considered when investigating environmental CWD prion contamination and indirect CWD transmission. Furthermore, most studies focused on prion strain characterization utilized mouse bioassay, where prion isolates are passaged into transgenic mice expressing PrP^C^ from another species. The resulting disease phenotype in the mouse and the biochemical characteristics of the prions from the brains of infected mice are assessed to give a set of disease characteristics that are then defined as a prion strain (18, 19). However, prion strains biologically cloned *via* mouse passage may be quite different than prions originally isolated from the natural host. Indeed, biological cloning of prions, by definition, results in stabilization of a set of biological and biochemical traits that may be different than those of the original isolates. Biochemical analyses of primary CWD prions isolated from different tissues in the same natural host are essential to understand critical features of prion strains and the diseases they cause. However, scant research so far has investigated thoroughly the biophysical and biochemical characteristics of prion strain isolated from different tissues within and among hosts before passaging these isolates into mice. In most cases, biologically cloning CWD strains by serial passage in the natural host is logistically and financially impossible. Here, we compare biochemical, conformational, and stability traits between LN and brain-derived prions within and among natural hosts before passaging into mice. Any differences we find may have important implications for horizontal, indirect, and even zoonotic CWD transmission and disease progression.

Of the paired samples received for analyses from nine deer, we obtained interpretable data from only four pairs of obex and LN tissues for intra-animal comparison (Table 1). We optimized our tissue preparation and Western blotting methods to obtain comparable, interpretable data, highlighting an important note about strain differences observed between the obex and LN samples. The central nervous system expresses the most PrP^C^, followed by lymphoid tissues and to a much lesser extent other peripheral tissues (8, 58, 59, 60, 61, 62, 63, 64, 65, 66, 67). PK may fail to fully digest high PrP^C^ levels in the brain to give accurate results if not more aggressively digested. Cervid PrP^Sc^ also may aggregate into denser plaques that protect cerPrP^C^, necessitating extra dilution, detergent, and/or PK for complete PrP^C^ solubilization and digestion from brain samples.

We had incomplete data for samples from deer 07416 and 10030 because we detected cerPrP^Sc^ only in LN, not obex samples. CWD prions often replicate to detectable levels in lymphoid tissues before the brain (8, 13, 17). So, these two brains likely contain too few prions, or perhaps nascent, protease-sensitive oligomeric prions that PK and WB fail to detect. Mouse bioassay could determine the prion titer, if any, and the biological characteristics of these prions, if present, in the brains of these two animals. Surprisingly, we detected cerPrP^Sc^ in obex and LN from a male deer aged just 1.5 years (10083). This rare occurrence of prions in the central nervous system (CNS) in this young cervid may indicate an early, perhaps vertical transmission event (6, 7, 68). We were less surprised to detect cerPrP^Sc^ in LN but not brains than our converse discovery. We detected cerPrP^Sc^ consistently in the brain of deer 14707, but inconsistently in the paired LN sample. Difficulty with tissue homogenization and Western blotting likely contributed to the inconsistent results with that sample. Finally, samples from two deer (10080 and 07415) did not yield interpretable data for either the brain or the LN. These samples likely had prion levels that were below the limit of detection by Western blot and were not able to be analyzed in this study.

For the four samples that we did have interpretable, reproducible data in both tissues (10023, 10074, 10083, and 07415), we observed no significant differences in the mean [GdnHCl]_1/2_ values between the obex- and LN-derived prion samples (Fig. 2), suggesting similar conformational stability of each isolate. We and others have shown that CWD prion strains are among the more conformationally stable prions, compared to mouse and hamster prions, for example (8, 49, 50, 64, 68). An emerging characteristic of CWD overall, then, seems to be relatively high conformational stability in the presence of GdnHCl (69, 70, 71, 72). These data presented here indicate that this increased stability in the presence of denaturing agents transcends tissue origin.

We did observe differences in electrophoretic mobility of the obex and the LN samples treated with ≥2.5 M GdnHCl that suggest more subtle differences in prion structural dynamics between prions present in these two tissues. All obex samples shift farther down the gel than LN samples when treated with 2.5 M or greater of GdnHCl, indicating that neurogenic prions adopt a unique conformation at these chaotrope concentrations, allowing PK differential access to these prions compared to lymphogenic prions. Whether these apparent biochemical differences between neurogenic and lymphogenic prions in conformational stability and structural dynamics translate to biological significance remains to be determined. We also detected numerous high-molecular weight species staining for PrP, which may represent prion oligomers, in both obex and LN samples.

While we observed no differences between mean [GdnHCl]_1/2_ values between prions isolated from LNs compared to obex, we witnessed more variable conformational stability in LN-derived prion samples, as evidenced by large variances observed at each [GdnHCl] (Fig. 2). Moreover, we observed unequal variances between the brain and LN mean [GdnHCl]_1/2_ value of one animal (10083) and a nearly significant difference in another (10074), indicating more variability in lymphogenic prion conformation. However, we did observe differences in the best-fit denaturation curves between brain and LN samples. Denaturation curves generated from LN samples exhibited far more variance, lower R^2^ values, and wider 95% CI bands than curves from brain samples. These data potentially reveal biochemical differences between the two prion sources that aren’t entirely represented in the sample means (Fig. 4).

We also found significant differences in glycoform ratios between the paired obex and LN-derived prions in all four animals tested. Glycoform ratio differences are an important indicator of different prion strains and another line of evidence that the prions present in the LN differ somewhat to those in the obex of the same animal. These differences could signify a biochemical strain difference between these animals; however, there is no systematic assessment of the glycosylation pattern of PrP^C^ in the LN or brain of white-tailed deer, and it is possible there are different pools of differentially glycosylated PrP between these two tissues. In rodent models of scrapie, investigations of PrP^C^ glycosylation profiles in the brain suggest that glycosylation influences neuroinvasion, PrP^Sc^ deposition and neuropathological lesion profiles (26, 73). Similar studies with mouse models of CWD or observational studies of infected deer could determine the biological relevance of our observed cerPrP^Sc^ glycosylation differences.

Samples from individual animals were then averaged together to assess any tissue differences between the obex and LN samples across animals. We found no statistical differences in conformational stability (Fig. 3), but the variances between the samples differed significantly. We also observed significant differences in glycoform ratios between brain and LN-derived cerprP^Sc^ (Fig. 5, Tables 2 and 3). These results mirrored the results observed in individual deer, reinforcing that biochemical strain differences exist between obex and LN CWD prions.

Lastly, we assessed biochemical strain differences among PrP^Sc^ present in the same tissue across individuals to identify any differences among animals. We observed no differences in conformational stability among any of the obex or LN samples across individuals. These data suggest that while there are some indications of conformational differences among strains between tissues, there do not appear to be any significant conformational differences between PrP^Sc^ in tissues across individuals. When we compared PrP^Sc^ glycoform ratios from the same tissue type across individual deer, we observed limited differences in glycoform ratio in the LN samples (Fig. 5 and Table 2). All significant differences occurred between sample 07416 and 10074 or 07399. Surprisingly, though, we observed many differences in obex PrP^Sc^ glycoform ratios among individual deer (Fig. 5 and Table 3).

Difficulties in lymphoid tissue preparation and low prion titers in these tissues may contribute to the variance we observed in the conformational stability and glycosylation profiles of lymphogenic prions. To address this, we employed a novel ELISA-based assay to probe structural differences among prions (44). The 7-5 ELISA specifically captures PrP and virtually eliminates background noise introduced by Western blotting. This approach allows us to assess differences in prion conformations in the central region of PrP that converts from predominantly alpha helical to beta sheet conformation, as well as the overall stability of that conformation. This technique again shows greater conformational variance in lymphogenic prions compared to neurogenic prions, which exhibited significant differences in prion conformation and stability among cervid brain prion isolates across individual deer. Structural profiling using a panel of antibodies scanning most of the PK-resistant prion core revealed consistent banding patterns among the lymphogenic prion samples but much greater variability in reactivity and banding patterns among neurogenic prions from individual deer. Interestingly, the D13 antibody did not react to any neurogenic prions, including our reference E2 brain isolate, but did react with lymphogenic prions, revealing a potential pool of prions in LNs not present in brains of these animals.

**Figure 8:**
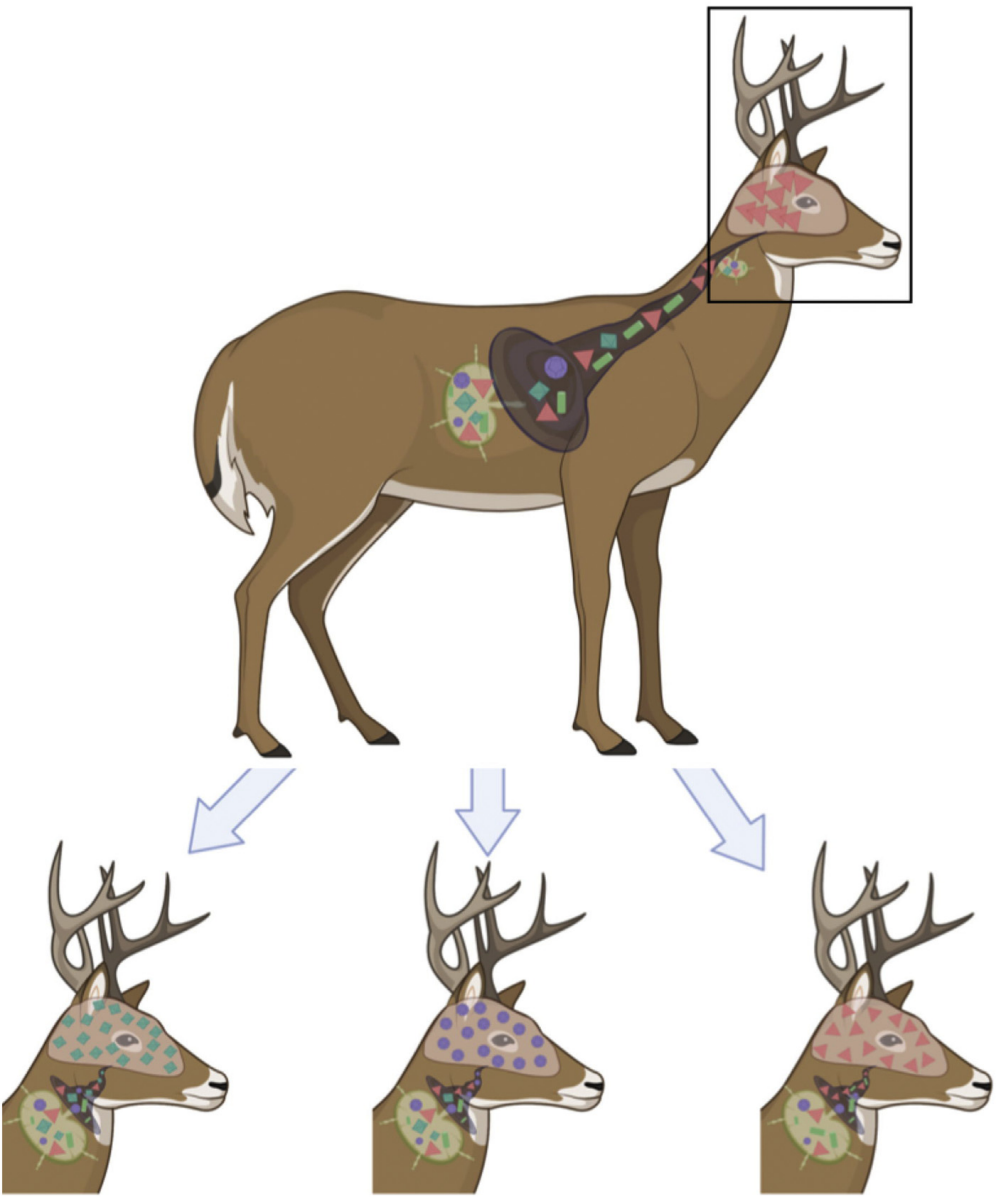
**Proposed model of lymphoid prion replication influencing differential neural prion selection and propagation in individual cervids.** Various lymphoid prion receptors, including Complement proteins CD21/35, Factor H, C3, and C1q, among others, select and replicate a more diverse pool of prions. PrP^C^, the predominant prion receptor in the CNS, selects a more restricted pool of prions, indicated by the narrowing “funnel” to the brain. These prions may differ significantly among individuals, resulting in cervids expressing different predominant strains, likely with variable zoonotic potential. CNS, central nervous system.

Based on our hypothesis of greater strain diversity of lymphogenic prions, we propose a model in which diverse prion strains present in the lymphoid organs may traffic to the brain, where different PrP^C^ glycoforms present in different neuroanatomical regions select specific cerPrP^Sc^ isoforms and propagate specific of prion strains in those regions (Fig. 8). This relatively more homogeneous group of prions produces predominant neurogenic prion strains that may be quite distinct among individual deer, like we observed in this study. The more variability that we see in LN prions may be due to a larger and more diverse population of prion strains in this extraneural site. We previously identified Complement proteins CD21/35 and Factor H as high-affinity prion receptors in extraneural sites (34, 35, 36, 37). Since the CNS express neither CD21/35 nor Factor H, but express far more PrP^C^ than the lymphoid system, PrP^C^ likely acts as the dominant prion receptor in the CNS and selects a more restricted set of prions, perhaps influenced by PrP^C^ glycosylation and more restricted templating of PrP^C^. Increased numbers and diversity of prion receptors outside the CNS may result in increased diversity of prion strains in extraneural tissues, as our biochemical data presented here indicates. Extraneural prions have been shown to have a wider species tropism that CNS prions (72). We propose that the increased prion diversity we measured biochemically in this work potentially increases their zoonotic potential as well.

Taken together, the data presented here provide strong evidence for biochemical strain differences between obex- and LN-derived prions in these cervids. Mouse bioassays are underway to see if these biochemical differences translate to biological differences and greater species tropism with zoonotic potential. If so, these results suggest that research should focus on extraneural prions and prion strains as they differ from neurogenic prions, are more likely to be shed into the environment, and expose other cervids and humans to prions with greater zoonotic potential. This proposed increased zoonotic potential of lymphogenic prions also has implications for best practices and policies regarding what tissues hunters and producers provide to agencies and what diagnostic tests those agencies perform on those tissues to assess not just prion positivity but also strain properties that may indicate zoonotic potential.

## Experimental procedures

### Sample homogenization

LN and obex samples from white-tailed deer that tested positive for CWD by ELISA performed in a National Animal Laboratory Network lab were provided frozen from the Arkansas Game and Fish Commission. Samples were stored at −20 °C until processing. We implemented several measures to minimize sample cross-contamination. Samples were trimmed with disposable scalpel blades on a half of a Petri dish. Both were discarded after a single use. Gloves and lab bench paper were also changed between each sample. LN samples were then placed in homogenizing tubes with 7 to 10 zinc zirconium homogenizing beads (2.3 mm diameter) and homogenized to 20% w/v in protein misfolding cyclic amplification (PMCA) I buffer (1x PBS 150 mM NaCl, 4 mM EDTA) with complete protease inhibitor (Roche). Samples were homogenized on a BeadBlaster for 10 rounds, with each round consisting of three cycles of a 30 s pulse at 6 m/s followed by a 10 s rest between each pulse. Samples were rested on ice for 5 min between each of the 10 rounds. Once samples were homogenized, samples were aliquoted and stored at −20 °C until further use. Obex samples were also processed to 20% w/v homogenate in PMCA I buffer and protease inhibitor as described above, but obex samples were homogenized with 7 to 10 glass beads (2.7 mm diameter) and for 2 to 3 rounds with a 5 min rest on ice between each round on the BeadBlaster. Samples were then aliquoted and stored at −20 °C until use.

### Prion titer determination

The cervid prion cell assay and PMCA were performed as previously described (44, 45). We used 10^−3^, 10^−4^, and 10^−5^ dilutions of brain samples and 10^−1^, 10^−2^, and 10^−3^ dilutions of LNs in each assay.

### Conformational stability assay and glycoform ratio

To assess the conformational stability of the prions from the brain and the LN, samples were thawed, and 15 μl of sample was added to 15 μl of GdnHCl in 0.5 M increments from 0 to 4 M, briefly vortexed, and incubated at room temperature for 1 h. After the 1 h denaturation, samples were precipitated in ice-cold methanol overnight at −20 °C. The following day, samples were removed from the −20 °C, centrifuged at 13,000 rcf for 30 min at 4 °C. Then, GdnHCl and methanol were removed, and the protein pellet was resuspended in either 18 μl of PMCA I buffer (LN samples) or 36 μl of PMCA conversion buffer (1x PBS 150 mM NaCl, 4 mM EDTA, 1% Triton-X 100, obex samples). LN samples then had 2 μl of 500 μg/ml of PK (Roche) (diluted in 1x PBS and 0.5 M EDTA) added for a final PK concentration of 50 μg/ml in each sample. Obex samples had 4 μl of 1000 μg/ml of PK (Roche) (diluted in 1x PBS and 0.5 M EDTA) added for a final PK concentration of 100 μg/ml. Samples were then incubated on a shaking heat block for 30 min at 37 °C and 800 rpm. Twenty microliters of each sample were denatured in the presence of 10 μl of 3x loading buffer (2.5 volumes of 4x sample loading buffer [Invitrogen] per one volume of 10x sample reducing agent [Invitrogen]) for 10 min at 95 °C. Samples were then either saved at −20 °C or immediately run by Western blot and analyzed for conformational stability and glycoform ratio.

### Western blotting

Samples were run on 12% bis-tris gels [NuPage] in 1x MOPS running buffer and transferred to polyvinylidene difluoride membranes. Nonspecific binding was reduced by blocking the membranes in 5% nonfat dry milk and 1% Tween-20 in 1x PBS (NFDM) for 1 h with rocking at room temperature. Membranes were then incubated in HRP-conjugated anti-PrP monoclonal antibody Bar224 (Cayman Chemical) diluted to 1:20,000 in SuperBlock (Thermo Fischer) overnight at 4 °C. Blots were washed the following day in PBST (0.2% Tween20 in 1x PBS) six times for 5 min each wash. Membranes were developed using enhanced chemiluminescent substrate (Millipore) for 5 min before imaging on ImageQuant LAS 4000 (GE).

### 7-5 ELISA

Conformation-based capture ELISA using capture antibody PRC7 and detection antibody PRC5 was performed as previously described (44) on tissue samples treated with increasing concentrations of GdnHCl.

### Data analysis

Densitometric analyses were completed in ImageJ. Conformational stability was determined by calculating the concentration at which the signal was half of the input ([GdnHCl]_1/2_) after fitting the data to a four-parameter linear regression curve in GraphPad Prism as previously described (71, 74, 75). Glycoform ratio was calculated in ImageJ by determining what percentage of the total signal was contributed by each glycosylation state. Glycoform ratio data were arcsine transformed before statistical analysis so percent data would fit a normal distribution. Only samples that had at least three successful replicates (conformational stability) or had results replicated on at least two blots with three samples each (glycoform ratio) were included for analysis. For the 7-5 ELISA, we calculated mean light absorbance measured at 450 nm for three independent experiments. Statistical analysis and graphing were performed in GraphPad Prism (version 8.30).

## Data availability

All data are contained within the manuscript. Raw data can be shared upon request to Dr Mark Zabel (mzabel@colostate.edu).

## Conflict of interest

The authors declare that they have no conflicts of interest with the contents of this article.
